# Acute Ischemic Stroke Due to Incomplete Endothelialization of a Left Atrial Appendage Occluder

**DOI:** 10.7759/cureus.40214

**Published:** 2023-06-10

**Authors:** Stacey Damito, Zhongying Liu-An, Haroon Faraz

**Affiliations:** 1 Internal Medicine, Hackensack University Medical Center, Hackensack, USA; 2 Interventional Cardiology, Hackensack University Medical Center, Hackensack, USA

**Keywords:** incomplete endothelialization, peri-device leak, stroke, watchman device, therapeutic anticoagulation, mitral regurgitation (mr), atrial fibrillation (af)

## Abstract

Left atrial appendage occlusion (LAAO) has become a preferred alternative for ischemic stroke prophylaxis in patients with a high risk of cardioembolic stroke but who are contraindicated for long-term anticoagulation. While the intervention has been successful in reducing bleeding events when compared to using anticoagulation, some stroke risk still persists. We present a case of stroke related to the failure of a left atrial appendage occluder, which was found to have a peri-device leak and incomplete endothelialization. In our case, we also believe these may have been exacerbated by comorbid severe mitral regurgitation. While current post-procedural protocols do address management of specific findings predictive of device failure, our patient still suffered from ischemic stroke despite following guidelines. Based on current outcome studies on LAAO, he may have been at higher risk than appreciated. His surveillance imaging at post-operative day 45 revealed a small peri-device leak of < 5 mm, which is now found to be associated with a higher embolic stroke risk than larger leaks of > 5 mm. Moreover, his mitral regurgitation, which was severe and borderline symptomatic, remained undertreated for a prolonged period. In cases of similar comorbidities, one might consider exploring the role of concomitant endovascular mitral repair and LAAO to optimize outcomes.

## Introduction

Stroke is the most common complication of atrial fibrillation (AF), with 90% of thrombi originating from the left atrial appendage (LAA) [1]. Stroke prevention remains the cornerstone of AF management. While warfarin and direct oral anticoagulants (DOAC) are currently the mainstay choices of therapy for stroke prophylaxis, long-term anticoagulation is often contraindicated in patients with high risks of bleeding. 

Left atrial appendage occlusion (LAAO) has emerged as a preferred intervention in such patients. Currently, there are two U.S. Food and Drug Administration-approved devices in the United States: Amplatzer Amulet (Abbott Medical) and Watchman/Watchman FLX (Boston Scientific) [2]. In a minimally invasive procedure, the occluding device is implanted into the LAA, where it seals off the ostium and prevents potential thrombi from embolizing into the systemic circulation. Afterward, patients are started on warfarin and aspirin. At post-procedural day 45, surveillance imaging (transesophageal echocardiogram [TEE] or cardiac computed tomography angiography [cCTA]) is performed to check for device-related complications. If there is evidence of adequate left atrial appendage (LAA) seal, a peri-device leak < 5 mm, and no device-related thrombus, anticoagulation is discontinued. Patients are then typically transitioned to dual antiplatelet therapy (i.e., clopidogrel and aspirin) for six months, followed by aspirin indefinitely [3]. Otherwise, patients continue anticoagulation with aspirin for at least six additional months until reassessment with repeat surveillance imaging.

## Case presentation

**Figure 1:**
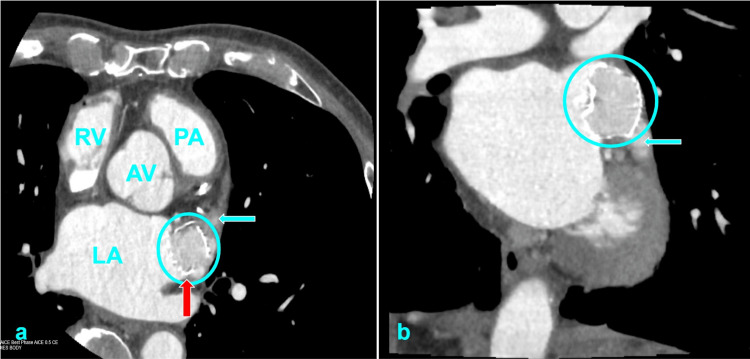
Cardiac computed tomography with contrast on surveillance imaging at day 45 after left atrial appendage occlusion (LAAO). Complete contrast opacification is noted within the LAAO device (blue circle) and left atrial appendage (LAA) (blue arrow in panels a & b), with a peri-device leak noted along the posterior edge of the LAA ostium (red arrow). Panel (a) shows the heart in the transverse plane and (b) in the coronal plane. LAAO: left atrial appendage occlusion, LAA: left atrial appendage, RV: right ventricle, LA: left atrium, AV: aortic valve, PA: pulmonary artery.

An 80-year-old man with moderate mitral regurgitation (MR) undergoes LAAO (Watchman FLX, 35 mm, Boston Scientific, Marlborough, MA). He was previously on apixaban for stroke prophylaxis in atrial fibrillation until it was discontinued due to recurrent hematuria. The CHA₂DS₂-VASc score is 4, and the HAS-BLED score is 4. After a successful LAAO, he resumes anticoagulation using apixaban, this time without any bleeding complications. At his follow-up visit on post-procedural day 45, he undergoes cardiac computed tomography (CCT) (Figure 1), which reveals evidence of incomplete endothelialization, including complete opacification of the LAA, a small peri-device leak (PDL) with a <5 mm gap, and two LAA accessory lobes unsealed by the device. Given these findings, he is transitioned off anticoagulation per current post-procedural guidelines and continues only with aspirin daily.

**Figure 2:**
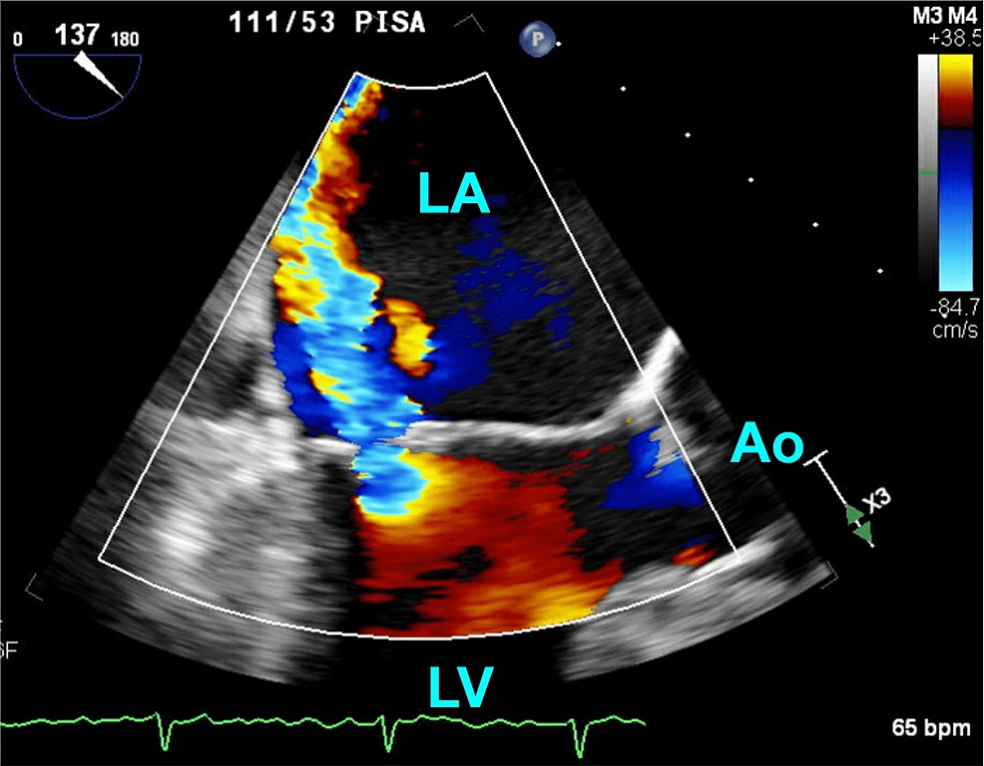
Midesophageal long view of the transesophageal echocardiogram at 10 months after Watchman placement. This was performed as part of the pre-operative evaluation for potential mitral valve repair. Color doppler showed a high-velocity, posteriorly directed mitral regurgitant jet ejecting retrogradely from the left ventricle. LV: left ventricle, LA: left atrium, Ao: aorta

**Figure 3:**
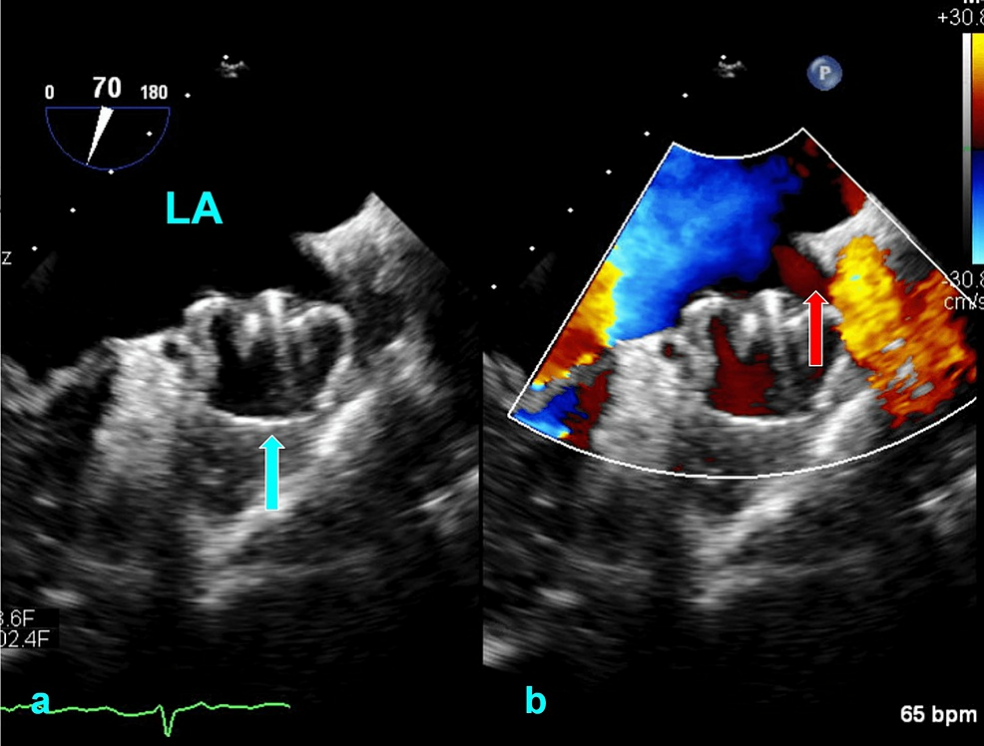
Right ventricle inflow-outflow view on the transesophageal echocardiogram (TEE) shows evidence of left atrial appendage occlusion (LAAO) failure at 10 months post-procedure. (a) TEE without color Doppler shows the Watchman device (blue arrow) seated in the left atrial appendage (LAA). (b) TEE with color Doppler shows evidence of flow around the Watchman device, indicating a peri-device leak (red arrow). LA: left atrium

He returns to be evaluated for mitral valve replacement 10 months later. TEE reveals a left ventricular ejection fraction of 62%, severe mitral regurgitation (effective regurgitant orifice 0.48 cm², regurgitant volume 75 ml) (Figure 2) with a posteriorly directed jet and a PDL that is now 6 mm (Figure 3). There is no evidence of a thrombus on the device’s atrial surface. There is no spontaneous echo contrast in the left atrium to suggest a patent foramen ovale.

**Figure 4:**
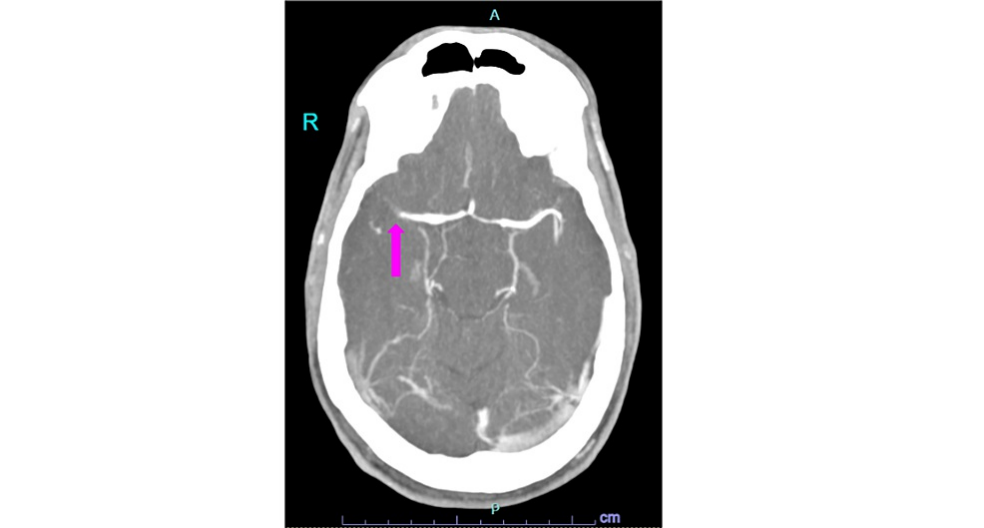
Computed tomography angiography of the head shows occlusion of the right middle cerebral artery M1 segment (pink arrow) R: right side of patient's head, A: anterior aspect, P: posterior aspect

Three days later, he presents to the emergency department with his wife, who reports that he has sudden-onset bilateral leg weakness and slurred speech. Vital signs show rate-controlled AF. Examination reveals an irregularly irregular heart rhythm, a 2/6 systolic murmur, and left hemiparesis. The initial NIHSS score is 4. Urgent head computed tomography confirms an acute ischemic infarct of the right middle cerebral artery M1 segment (Figure 4). Eligible for mechanical thrombectomy, he undergoes the procedure with good tolerance and successful reperfusion with thrombolysis in cerebral ischemia score of 3.

**Figure 5:**
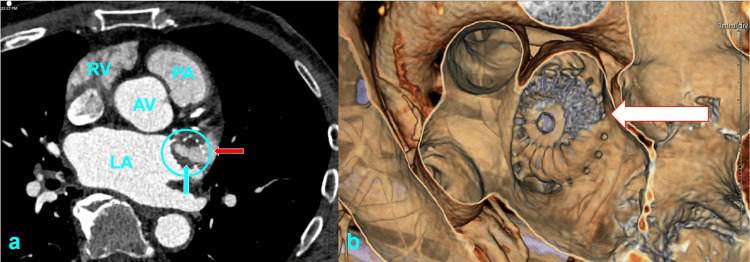
Cardiac computed tomography at 10 months after left atrial appendage (LAA) occlusion. The left panel (a) shows the heart in the transverse plane. Hypodense regions (blue arrow) consistent with thrombus are visualized alongside hyperdense contrast (red arrow) within the LAA occluder (blue circle) and appendage, indicating the presence of a device leak. Right panel (b) is a 3D rendering of the CT, which shows the atrial surface of the LAA occluder device (Watchman FLX) with only partial endothelialization (white arrow). RV: right ventricle, LA: left atrium, AV: aortic valve, PA: pulmonary artery

There is a concern for LAAO failure. Structural CCT (Figure 5) reveals incomplete endothelialization of the device’s atrial surface and contrast opacification within the LAA lumen consistent with device leak. A decision is made to restart anticoagulation using rivaroxaban. The patient’s neurological deficits resolve, and there are no bleeding complications. Once he stabilized, discussions regarding the repair of his mitral regurgitation and device failure were deferred to outpatient follow-up. Per his request, he was initially managed medically for his mitral regurgitation. Due to the progression of symptoms, he was eventually referred for mitral valve repair within two months. He underwent mitral valve clipping with symptomatic improvement. Due to only mild clinical improvement, he continues to be monitored for potential valve replacement and LAAO device repair.

## Discussion

LAAO has become a preferred alternative for stroke prophylaxis in patients contraindicated for long-term anticoagulation. This is attributed to the two landmark PROTECT-AF [4] and PREVAIL [5] trials, supported by the subsequent EWOLUTION [6], CAP, and CAP2 studies. In a five-year outcome meta-analysis, LAAO was significantly associated with reducing major non-procedural bleeding (hazard ratio [HR] 0.48, p = 0.0003), disabling/fatal stroke, hemorrhagic stroke, cardiovascular death, and all-cause mortality [6]. However, non-inferiority was not achieved in its prevention of all-cause (HR 0.95, p = 0.87) and ischemic stroke (HR 1.71, p = 0.08) [7], the most important goals of LAAO. While ~83% reduction is now demonstrated in real-world studies [7], the risk persists.

Current post-LAAO treatment guidelines are based on canine studies on the temporal nature of device neoendothelialization [8]. As described, the first three days post-implantation are characterized by fibrin deposition, sealing gaps in and around the device, followed by thrombus organization, resorption, and granulation via the inflammatory process until day 45. By day 45, endothelialization of the device’s atrial surface finally begins. By day 90, the new endocardial surface is expected to have sealed off the former LAA ostium and formed a contiguous surface with the adjacent atrial wall. This is the foundation on which anticoagulation protocols have been developed, but few human studies have been conducted to reinforce it.

Incomplete device endothelialization noted on surveillance imaging, while increasingly recognized, remains underinvestigated. While most patients fare well on current protocols, the presence of PDL, especially small (< 5 mm), at one-year post-implantation has been significantly associated with a higher five-year risk of ischemic stroke and systemic embolism (HR: 1.94; 95% CI: 1.15-3.29; P = 0.014) [9]. Another study showed that surveillance cardiac computed tomography angiography found incomplete endothelialization in 61% of patients by 10 ± 6 months [10]. Given the high incidence of incomplete device endothelialization that is markedly past the expected 45-day mark post-procedure, neoendothelialization may not be as consistent or as brief as previously expected. Studies on both device endothelialization in humans and contributing factors (like innate healing competency, internal hemodynamic disturbances, and structural nuances) are needed to guide evolving protocols on device surveillance, maintenance, and post-procedural therapies. 

Our patient had a few risk factors that may have contributed to the device’s failure. It should first be noted that imaging revealed no observable device-related thrombus or patent foramen ovale, lowering suspicion for embolic etiology at these sources. For this patient, one risk factor was his mild PDL. Such a gap inherently hinders complete endothelialization due to the limited juxtaposition of the device with the native endocardium. However, per guidelines, his small PDL did not require intervention. Given that small PDLs are associated with an increased risk for stroke [9,11], this now merits consideration for correction. Secondly, the presence of severe MR may have posed a few obstacles. One similar case of device failure involving severe MR suggested that a high-velocity, posteriorly directed jet could produce persistent shearing forces that retard tissue remodeling and unseat the device [12]. In the patient's initial TEE, a jet was noted to flow briskly against the same area indicated in Figure 3b. Given that this was at the lateral edge of the device, the pathophysiology is fathomable. Furthermore, our patient had a prolonged delay in repairing MR, which potentiated opportunities not only for these complications but also for progressive structural changes, especially left atrial, that exacerbated PDL. Lastly, while MR may be protective against thrombus formation in AF [13], there is a paradoxical disadvantage: thrombus is a critical substrate for endothelialization. This poses an obvious conflict in which thrombus is simultaneously favored and undesired. As it follows, mitral regurgitation may have a larger role in the success of LAAO than is recognized. 

In a case series, patients with comorbid MR and AF underwent a combined percutaneous procedure for mitral valve repair and LAAO, which resulted in overall clinical and valvular improvement and a hemodynamically insignificant left-to-right atrial shunt [14]. For patients in whom MR and AF are profound, simultaneous intervention may be merited. A one-time concomitant procedure offers significant advantages, including the reduction of clinical burden for the patient (e.g., frequent surveillance, psychosocial, and financial) and procedural redundancy for the interventionalist.

## Conclusions

LAAO has been a favorable alternative for stroke prophylaxis in patients with AF who are contraindicated for anticoagulation. However, PDL and incomplete endothelialization are critical complications for which guidelines need to elaborate, especially for those who are found to have PDL < 5 mm. In patients with mitral regurgitation, a concomitant intervention with LAAO may help further reduce stroke risk and overall healthcare burdens. As long-term outcome studies continue to inform post-LAAO complications, an evolving post-treatment protocol is expected and appreciated.

## References

[1] Regazzoli D, Ancona F, Trevisi N (2015). Left atrial appendage: physiology, pathology, and role as a therapeutic target. Biomed Res Int.

[2] (2023). FDA: LAAO adverse procedural outcomes potentially more common in women. https://www.fda.gov/medical-devices/letters-health-care-providers/left-atrial-appendage-occlusion-laao-devices-potentially-associated-procedural-outcome-differences#:~:text=The%20device%20mechanically%20occludes%20the,Watchman%20and%20Watchman%20FLX%20devices)..

[3] Magdi M, Renjithal SL, Mubasher M (2021). The WATCHMAN device and post-implantation anticoagulation management. A review of key studies and the risk of device-related thrombosis. Am J Cardiovasc Dis.

[4] Reddy VY, Sievert H, Halperin J (2014). Percutaneous left atrial appendage closure vs warfarin for atrial fibrillation: a randomized clinical trial. JAMA.

[5] Holmes DR Jr, Kar S, Price MJ (2014). Prospective randomized evaluation of the Watchman Left Atrial Appendage Closure device in patients with atrial fibrillation versus long-term warfarin therapy: the PREVAIL trial. J Am Coll Cardiol.

[6] Boersma LV, Ince H, Kische S (2019). Evaluating real-world clinical outcomes in atrial fibrillation patients receiving the watchman left atrial appendage closure technology: final 2-year outcome data of the EWOLUTION trial focusing on history of stroke and hemorrhage. Circ Arrhythm Electrophysiol.

[7] Reddy VY, Doshi SK, Kar S (2017). 5-year outcomes after left atrial appendage closure: from the PREVAIL and PROTECT AF trials. J Am Coll Cardiol.

[8] Schwartz RS, Holmes DR, Van Tassel RA (2010). Left atrial appendage obliteration: mechanisms of healing and intracardiac integration. JACC Cardiovasc Interv.

[9] Dukkipati SR, Holmes DR Jr, Doshi SK (2022). Impact of peridevice leak on 5-year outcomes after left atrial appendage closure. J Am Coll Cardiol.

[10] Granier M, Laugaudin G, Massin F (2018). Occurrence of incomplete endothelialization causing residual permeability after left atrial appendage closure. J Invasive Cardiol.

[11] Perkins J, Bhagat R, Nichols M, Shah J (2020). Reoccurrence of stroke in a patient with peri-device leak of WATCHMAN device. J Investig Med High Impact Case Rep.

[12] Batnyam U, Tuluca A, Witzke CF, Greenspan AM, Mainigi SK (2021). Failure of complete endothelialization of a watchman device 3 years post-implantation. JACC Case Rep.

[13] Bisson A, Bernard A, Bodin A, Clementy N, Babuty D, Lip GY, Fauchier L (2019). Stroke and thromboembolism in patients with atrial fibrillation and mitral regurgitation. Circ Arrhythm Electrophysiol.

[14] Francisco AR, Infante de Oliveira E, Nobre Menezes M, Carrilho Ferreira P, Canas da Silva P, Nobre Â, Pinto FJ (2017). Combined MitraClip implantation and left atrial appendage occlusion using the Watchman device: A case series from a referral center. Rev Port Cardiol.

